# Transitioning to competency-based medical education: impact of educational interventions on internal medicine residents’ understanding of the purpose and process

**Published:** 2019-11-28

**Authors:** Vijay J Daniels, Jesse Stach, Gurtej Sandhu

**Affiliations:** 1 Division of General Internal Medicine, University of Alberta, Alberta, Canada; 2 Internal Medicine Residency Program, University of Alberta, Alberta, Canada

## Background/purpose

Postgraduate medical education training in Canada has been in a transition from a purely time-based model to a more competence-based model. The new model places greater emphasis on residents demonstrating competence in the essential skills of their future profession, a model known as Competency-Based Medical Education (CBME), than on time spent in the program. In Canada, Family Medicine residency training was the first to transition implementing the College of Family Physicians of Canada’s Triple C Competency Based Curriculum.^1^

More recently, the other 66 specialties have begun their transition to the Royal College of Physicians and Surgeons’ Competence By Design^2^(CBD) model in a staged fashion with seven cohorts transitioning between 2017 to 2023. Core Internal Medicine residency training was part of the 2019 cohort, but the program at the University of Alberta transitioned to a CBME model early, with a pilot in July 2016 and launch in July 2017, two years before most other Canadian programs. In July 2018, we made the last changes to meet the Royal College’s CBD requirements of adding milestones to our EPA forms. The purpose of this paper is to describe our approach to engaging residents about CBME, to demonstrate the effectiveness of our approach, and to share resources we developed between July 2016 and July 2018 that may help the over 50 other specialties across the country launch in the coming years.

## Our approach

In July of 2016 we adapted our Mini-CEX forms to use entrustment language and mapped them to the draft version of the Entrustable Professional Activities (EPAs) available from our specialty committee. We asked residents to attempt one EPA observation per week, but few residents were able to get the requested one EPA per week. There were various reasons offered.

To address this, we conducted a needs assessment in January 2017 in which we surveyed our residents (56 of 99 responding), asking them to rate their agreement to statements regarding their understanding of the purpose and processes of CBME, and their understanding of EPAs and process to acquire them. Comment boxes were provided for residents to elaborate on any barriers. Our research ethics board approved these data collection procedures as well as a follow-up survey. The results indicated a few areas that needed to be targeted. First, residents did not fully understand what an EPA was and which to get at any given time. Second, residents felt they and their preceptors were unsure of their role in CBME, i.e. who is driving this process. Finally, residents did not understand how CBME was being operationalized, specifically, how individual assessments would be used to sign off EPAs and allow resident progress through stages.

To address these, we took three approaches. First, we created a series of short 5-10-minute online videos explaining the purpose of CBME including the roles of various stakeholders, what a competency-based assessment framework looks like, and approaches to planning which EPAs to get and how to ask preceptors for them. We used some of these videos in a Grand Rounds presentation to help front line preceptors prepare for July 1. The videos are available at the following link: https://tinyurl.com/UofACIMCBMEvideos

Second, we updated our Internal Medicine residents’ website with information regarding which rotations are high yield for which EPAs with information presented by EPA, and by rotation. Finally, we created a one to two-page EPA Quick Reference document for each of the four stages of training, so residents could see a high-level overview at a glance of the EPAs for a given stage. Screenshots of our website, the EPA Quick Reference documents, and the Grand Rounds presentation are available here: https://tinyurl.com/UofACIMCBMEresources

## Evaluation of initiatives

In fall of 2017, three months into a new academic year, we repeated our previous survey with 68 of 99 residents responding. We had success with improving residents’ knowledge of their role in CBME (medium effect size), and their perception of their preceptors’ understanding of CBME (medium effect size), but not their preceptor’s understanding of their role in CBME (non-significant increase). Residents had a better understanding of what EPAs were (large to very large effect size), and how assessments were used to sign off EPAs (medium to large effect size), but unfortunately, we were unable to improve resident’s knowledge of which EPAs to get or identifying good opportunities to get them. See Table 1.

**Table 1:** Resident responses to pre and post surveys

Scale	Pre-Survey Mean^*^	Post-Survey Mean^*^	Pooled SD	△ p-value	Effect size (Cohen d)
Knowledge of their role	4.89	5.48	1.24	0.012	0.47
Preceptors’ understanding of CBME	4.45	5.05	1.20	0.008	0.50
Preceptor’s understanding of their role in CBME	4.21	4.57	1.24	0.128	N/A
Understanding of EPAs	4.68	5.92	1.28	<0.001	0.97
How Assessments are used to sign off EPAs	4.66	5.47	1.24	0.001	0.66
Which EPAs to get	4.86	4.65	1.43	0.439	N/A
Having trouble identifying opportunities to get EPAs	5.21	5.05	1.60	0.581	N/A

* 1=Strongly Disagree, 4= Unsure, 7=Strongly Agree.

The comments indicated that the largest barriers were busy clinical services in which residents did not feel they could ask preceptors to take the time to fill out the EPA form, and frequent situations where the residents reviewed with a senior resident/subspecialty fellow and not the attending physician. Another barrier was that our new assessment system (which was adapted from our medical school’s locally developed system for our July 2017 launch) did not have a search function, so residents had to know which EPA covered, for example, Breaking Bad News and often selected a few EPAs before finding the correct one.

We asked residents to rate how helpful they found each resource on a Likert scale (1=Not helpful, 2=Slightly, 3=Somewhat, 4=Very, 5=Extremely). Residents found all resources somewhat to very helpful with the videos rated 3.6 (SD 1.0), updates to the website 3.6 (SD 0.8), and the EPA Quick Reference guides 3.7 (SD 0.8).

Since this last survey, we have developed some other videos to help residents and preceptors understand the stages of discipline specific to our specialty, as well as other tips to help streamline the EPA form process. Our videos have been shared with our postgraduate medical education office, which oversees all residency programs at the University of Alberta, and they have begun using the videos to help the other nine programs which have launched CBME in July 2017 and July 2018. As of September2019, based on the individual view count of the 11 videos we created, there have been1817 views.

## Summary

The resources we have developed have helped our residents’ transition to CBME and have begun helping other residency programs locally. There is still work to be done in terms of optimizing our assessment system to meet the needs of residents with features like a search function, and we need other strategies to help faculty understand their role in CBME.
